# The influence of calcitriol and methylprednisolone on podocytes function in minimal change disease in vitro model

**DOI:** 10.1038/s41598-023-39893-x

**Published:** 2023-08-05

**Authors:** Kamil Grubczak, Aleksandra Starosz, Barbara Makowska, Zuzanna Parfienowicz, Magdalena Krętowska, Beata Naumnik, Marcin Moniuszko

**Affiliations:** 1 Department of Regenerative Medicine and Immune Regulation, Medical University of Białystok, Jerzego Waszyngtona 13, 15-269 Białystok, Poland; 2 Ist Department of Nephrology and Transplantation with Dialysis Unit, Medical University of Bialystok, Żurawia 14, 15-540 Białystok, Poland; 3 Department of Allergology and Internal Medicine, Medical University of Białystok, Marii Skłodowskiej-Curie 24A, 15-276 Białystok, Poland

**Keywords:** Kidney, Nephrology

## Abstract

Minimal change disease (MCD), considered one of the major causes of nephrotic syndrome, is a complex pathological condition with disturbances in podocytes’ foot processes. Numerous studies suggested the essential role of vitamin D3 in maintaining proper glomerulus function. However, the data on direct potential of that compound in reference to podocytes are scarce. Thus, here we assessed the influence of calcitriol (active vitamin D3) on podocyte function, apart from commonly used steroids (methylprednisolone). CIHP-1 podocyte cell line was used to implement the LPS-PAN-induced MCD in vitro model. Viability, podocyte-related slit diaphragm proteins, morphology, function as a barrier was evaluated using flow cytometry, RT-PCR, confocal microscopy, and TEER analysis. Calcitriol or methylprednisolone did not affect cell viability. Podocyte-related proteins demonstrated different responses to in vitro treatment compared to previously reported changes in total glomeruli. Podocyte morphology was partially restored in the presence of the tested compounds. In addition, TEER analysis revealed improvement of LPS-PAN-induced cells' function as a barrier when vitamin D3 or steroid was used. In conclusion, a significant potential for modulation of MCD in vitro model podocytes with calcitriol or selected steroids was reported. Further studies on vitamin D3 in context of podocyte-related phenomenon accompanying MCD are of great importance.

## Introduction

Minimal change disease (MCD) is a pathological condition of glomeruli with complex etiology and subtle or no visible changes in a routine histological examination, affecting predominantly children (1–7 years). MCD is considered one of the major causes of nephrotic syndrome in children, characterized by proteinuria, hypoalbuminemia, peripheral edema, hyperlipidemia, lipiduria, and hypercoagulability. Unfortunately, the mentioned symptoms are not specific to MCD; especially in the early stage, the disease can even be asymptomatic. Interestingly, despite various symptoms of filtration barrier damage, the function of the kidneys is preserved in most cases. Treatment of MCD is based mainly on steroid drugs presenting a positive response to the therapy, even in long-term outcomes^1^. Even around 90% of children demonstrate a good response to applied treatment. In adults, the incidence of steroid resistance is higher and oscillates about 10–40%. In addition, the course of the disease in adult patients is often associated with nonselective proteinuria and renal failure. Unfortunately, most of these patients will not get complete remission but have poor long-term prognoses, leading to end-stage renal disease^2^.

Podocytes are highly specialized glomerular epithelial cells that cover the exterior surface of glomeruli with decisively differentiated morphological and functional properties. One of the podocytes' most distinctive morphological properties is the presence of characteristic foot processes expanded to the basement membrane of the capillaries with a filtration slit diaphragm between them. These unique cell junctions corresponded to the filtration processes' selectivity and the filtration barrier's proper function in glomeruli^3^. Capillary endothelial cells and the glomerular basement membrane (GBM) are involved in controlling the filtration processes in the Bowman capsule. Additionally, podocytes are also responsible for the synthesis of GMB components. Numerous reports have demonstrated a direct association of podocytes’ function and morphology with pathological conditions in kidney structure leading to glomerulosclerosis^4^. Furthermore, a wide range of proteins is reliable for podocytes' proper functional and structural properties, including nephrin (NPHS1), podocin (NPHS2), and others^5^.

Nephrin (NPHS1) is a transmembrane adhesion protein expressed in glomerular podocytes, constitutes an integral part of podocytes, and is the main component of slit diaphragm between adjacent foot processes. Together with podocin (NPHS2), nephrin is pending a crucial role in maintaining the selective permeability of the glomerular filtration barrier. Additionally, nephrin is necessary for the adequate development of glomeruli which was proved by Verme et al. using a knock-out mice model with a deletion in the *Nephrin* gene^6^. Phosphotyrosine/tyrosine phosphatase receptor type O (PTPRO), previously known as glomerular epithelial protein 1 (GLEPP1), is crucial in regulating podocyte morphology, and its reduced expression can result in overall shortening of slit diaphragm^7^. Additionally, tight junctions maintain cell patency to regulate foot process structure. In general, glomerulonephropathies are associated with decreased levels of PTPRO and its heterogeneous distribution in glomeruli, especially in conditions such as IgA nephropathy (IgAN). In addition, these diseases were also associated with diffused effacement of podocyte foot processes^8^. Another essential podocyte-related protein is podocalyxin (PODXL), the main glycocalyx component localized to the apical cell of glomerular podocytes. PODXL maintains the podocyte’s shape and slit diaphragm together with previously mentioned nephrin and podocin. Due to negative membrane potential, adjacent foot processes are kept separated. Loss of structural integrity has been found to lead to various glomerular diseases to which the abnormal podocalyxin may contribute in most cases^9^. Moreover, podocalyxin was reported at high concentrations in the urine of patients with preeclampsia, and more importantly, correlating strongly not only with proteinuria but also with nephrin expression^10^.

Considering a wide range of immunomodulatory properties, tremendous potential is currently seen in the involvement of vitamin D receptor in novel therapeutic applications^11^. Most attention is focused on its possible protective role in the course of chronic inflammation, including renal impairment^12^. Administration of calcitriol or its analog is obligatory in severe stages of chronic kidney disease, considering that compartment being the main source of its generation^13^. Vitamin D3 has been demonstrated to be a crucial element in maintaining kidney filtration homeostasis by preserving the structural integrity of the slit diaphragm on podocytes^14^. Moreover, calcitriol was found to preserve damage to tight-junction proteins and nephrin in rats with membranoproliferative GN^15^. Despite numerous research, the direct effects of vitamin D3 on podocytes have not been thoroughly investigated. It is well known that the role of calcitriol—an active form of vitamin D, is not only limited to the modulation of calcium and phosphorus concentrations. Wide range of immunomodulatory properties of vitamin D3 has been shown in reference to nephropathy, including inter alia: anti-inflammatory, anti-fibrotic, and podocyte protective effects^16,17^. In the diabetic nephropathy rat model, calcitriol use demonstrated favorable effects on kidney function, with reduced albuminuria and attenuated lesions^18^. However, questions regarding the mechanism of these actions still remain unresolved.

Minimal change disease can lead to kidney damage, and the lack of specific markers used in routine diagnostics caused that detection usually occurs in the presence of extensive changes. Considering that, we decided to evaluate the in vitro model of podocyte damage corresponding to MCD. Additionally, our study also investigated potential methods of supporting the treatment by using the immunomodulatory properties of Vitamin D3 and steroid approach to the molecular pattern of slit diaphragm proteins.

## Results

### Viability of MCD model podocytes treated with active vitamin D3 or methylprednisolone

**Figure 1:**
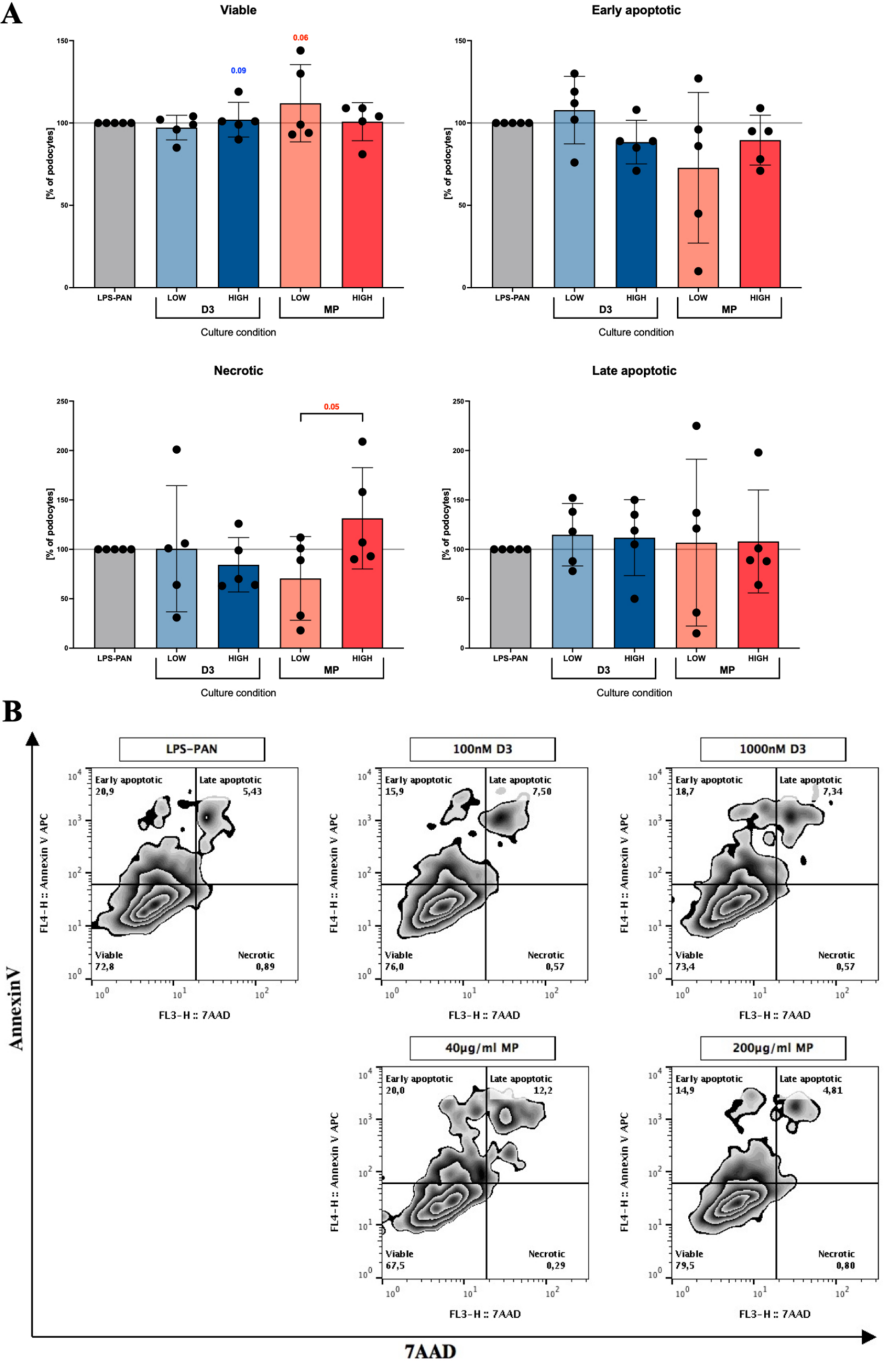
Analysis of podocytes viability and surface continuity in culture with vitamin D3 or steroid. Changes in frequency of viable, early apoptotic, late apoptotic and necrotic podocytes cultured for 48 h in presence of a low and high concentration of vitamin D3 (D3; LOW = 100 nM, HIGH = 1000 nM) and methylprednisolone (MP; LOW = 40 μg/ml, HIGH = 200 μg/ml) (**A**). Sample flow cytometric zebra plots from podocytes viability analysis using Annexin-V FITC and 7-AAD (**B**). Data are presented as a mean value with standard deviation (mean ± SD) (*n* = 5). The levels of significant differences were indicated with asterisks or exact p values: *p < 0.05; **p < 0.01; ***p < 0.001; ****p < 0.0001.

We did not report the unfavorable influence of tested concentrations of vitamin D3 or methylprednisolone on the viability of podocytes. In contrast, we found that vitamin D3 at 100–1000 nM or methylprednisolone at 40–200 μg/ml rather support the survival of podocytes in the MCD model. These findings could be supported by a tendency for higher frequencies of viable podocytes (AnnexinV−7AAD−) and a reduction in necrotic cells. Noteworthy, the MCD induction process did not affect any viability-related parameter (Fig. 1; Supp. Fig. S1A).

### Alterations in podocyte-related selected proteins in response to in vitro treatment with active vitamin D3 or methylprednisolone

**Figure 2:**
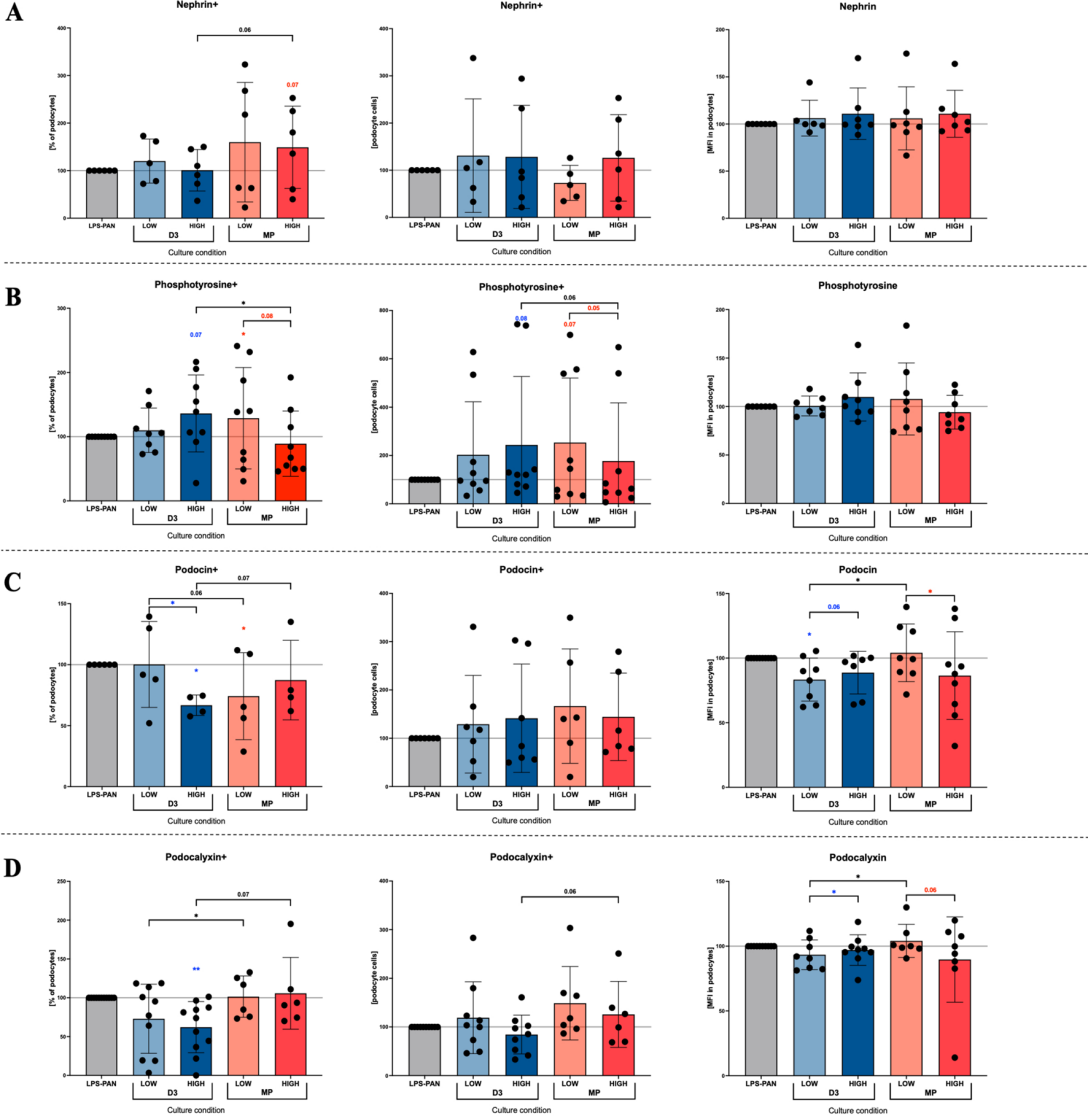
Assessment of selected podocyte-related proteins in culture with vitamin D3 or steroid. Low and high concentrations of vitamin D3 (D3; LOW = 100 nM, HIGH = 1000 nM) and methylprednisolone (MP; LOW = 40 μg/ml, HIGH = 200 μg/ml) were tested on podocytes cultured for 48 h to analyze changes in nephrin (**A**), phosphotyrosine (**B**), podocin (**C**) and podocalyxin (**D**). Protein variations were measured as a frequency or the total number of cells expressing selected marker and mean fluorescence intensity (MFI) within podocytes. Data are presented as a mean value with standard deviation (mean ± SD) (*n* = 7). The levels of significant differences were indicated with asterisks or exact p values: *p < 0.05; **p < 0.01; ***p < 0.001; ****p < 0.0001.

In the context of podocyte-related proteins essential in maintaining proper glomeruli filtration, we found a tendency for a higher frequency of nephrin-positive podocytes in the presence of a high concentration (200 μg/ml) of methylprednisolone. Noteworthy, such opposite to vitamin D3 direction of changes was close to statistical significance. In addition, analysis of an absolute number of the cells with that marker or its mean expression within single cells was not reported in both, steroid and vitamin D3 treated samples (Fig. 2A). Phosphotyrosine analysis revealed a higher frequency and an absolute number of podocytes expressing that protein when treated with vitamin D3 at the highest concentration of 1000 nM. Interestingly, only lower concentrations of methylprednisolone led to an increase in phosphotyrosine-positive cells frequency and cell number, with a decline toward initial values at the highest concentration used. Therefore, significant dominance of cells expressing phosphotyrosine was observed in samples subjected to vitamin D3 compared to steroids, at 1000 nM and 200 μg/ml, respectively (Fig. 2B). Methylprednisolone was found to reduce the frequency of podocin-positive podocytes in the MCD model in a dose-dependent manner. Vitamin D3 reduced podocin-positive podocytes at a concentration of 100 nM with no further decline at 1000 nM. On the contrary, the reduction of podocin expression within cells was more constant in the presence of vitamin D3 podocytes, with alternating decrease and increase towards initial values in steroid-treated samples (Fig. 2C). Changes in podocalyxin were predominantly observed in podocytes subjected to vitamin D3, with decreased values in the context of frequency and cellular expression. Because the MCD model demonstrated higher podocalyxin levels, vitamin D3 caused a beneficial reduction in unstimulated podocyte values. Methylprednisolone seemed to reduce podocalyxin; however, these values were less pronounced compared to vitamin D3 and did not reach a significance level (Fig. 2D) (Supp. Fig. S1B).

### Expression of selected podocyte-related mRNA in vitamin D3- or steroid-treated podocytes

**Figure 3:**
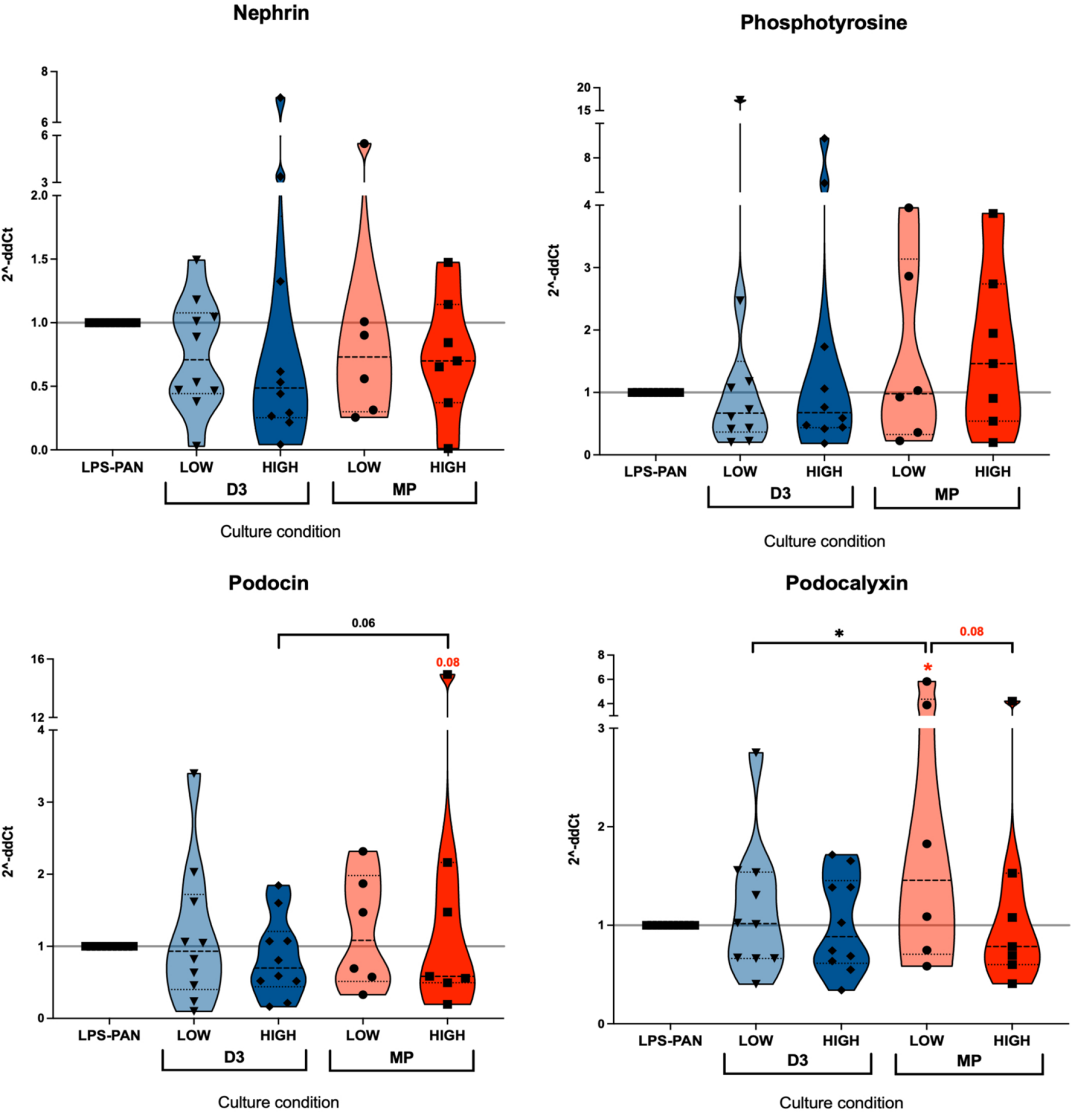
Evaluation of podocyte-related proteins expression on mRNA level within podocytes treated with vitamin D3 or steroid. Different concentrations of vitamin D3 (D3; LOW = 100 nM, HIGH = 1000 nM) and methylprednisolone (MP; LOW = 40 μg/ml, HIGH = 200 μg/ml) were applied to podocytes in 48-h culture. Assessed genes included those coding: nephrin (*NPHS1*), phosphotyrosine (*PTPRO*), podocin (*NPHS2*), and podocalyxin (*PODXL*). Data are presented as a mean value with standard deviation (mean ± SD) (*n* = 7). The levels of significant differences were indicated with asterisks or exact p values: *p < 0.05; **p < 0.01; ***p < 0.001; ****p < 0.0001.

Preliminary tests investigating the generation of MCD podocytes demonstrated results supporting protein level analyses. In accordance, we found significantly higher expression of *PODXL* (podocalyxin) in podocytes subjected to in vitro LPS-PAN therapy. Simultaneously, the same samples seemed to demonstrate slightly lower expression of NPHS2 (podocin) and PTPRO (phosphotyrosine) in the MCD model compared to unstimulated cells. Despite significant changes in protein level in the context of nephrin and phosphotyrosine, the mRNA level of these proteins—*NPHS1* and *PTPRO,* respectively, were comparable to MCD podocytes alone after 48 h. Similar results were observed in reference to *NPHS2* and *PODXL*; however, the difference between vitamin D3 and methylprednisolone effects was maintained with higher expression in steroid-treated cells (Fig. 3.; Supp. Fig. S1D).

### Cytokine profile assessment in MCD model podocytes treated with vitamin D3 or steroid

**Figure 4:**
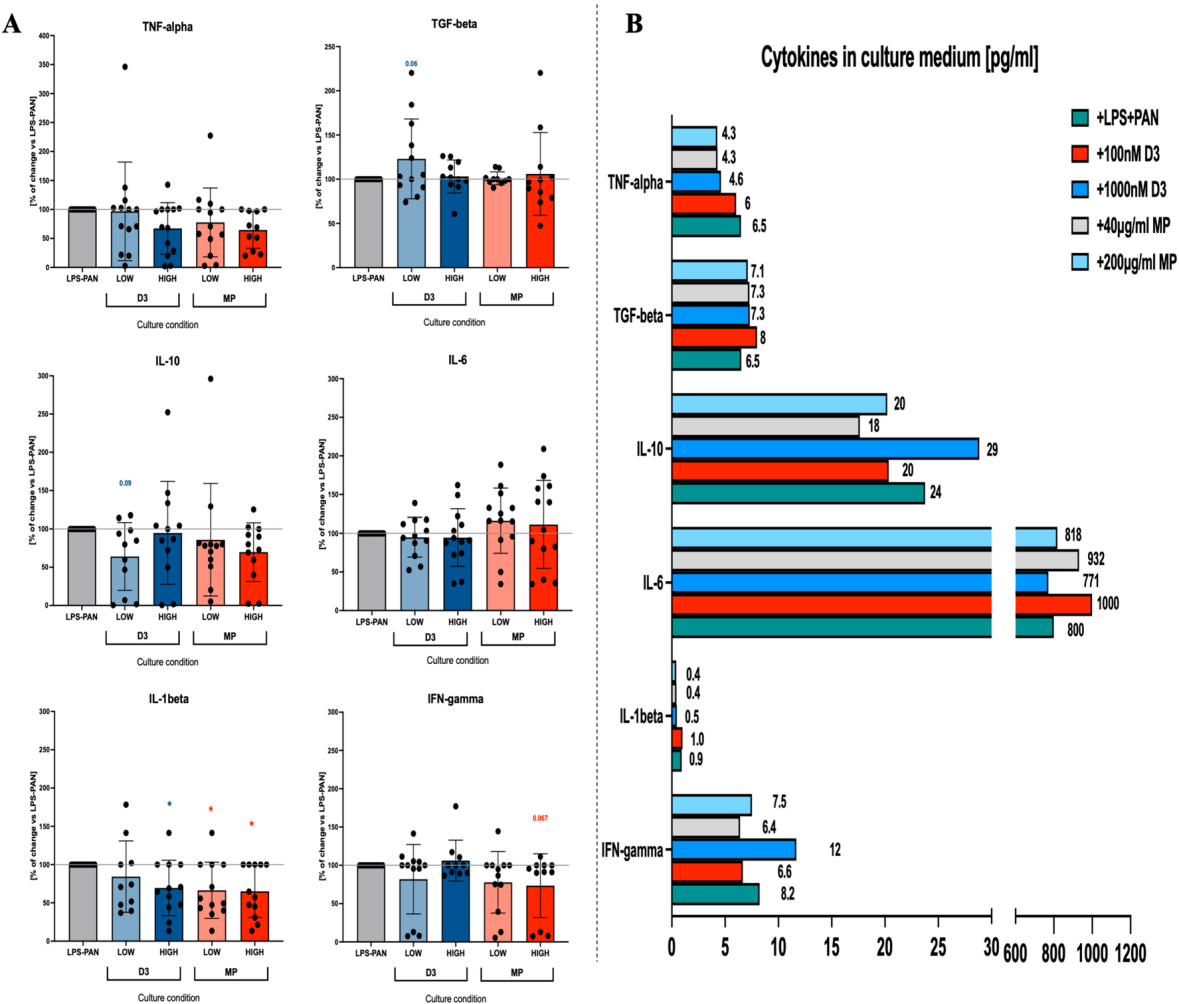
Analysis of selected cytokines release profile in the culture of podocytes treated with different concentrations of vitamin D3 or steroid. Different concentrations of vitamin D3 (D3; LOW = 100 nM, HIGH = 1000 nM) and methylprednisolone (MP; LOW = 40 μg/ml, HIGH = 200 μg/ml) were applied to podocytes in 48-h culture. Data presented as frequency of change relative to the LPS-PAN stimulation TNF-alpha, TGF-beta, IL-10, IL-6, IL-1beta, and IFN-gamma. Data are presented as a mean value with standard deviation (mean ± SD) (*n* = 10). The levels of significant differences were indicated with asterisks or exact p values: *p < 0.05; **p < 0.01; ***p < 0.001; ****p < 0.0001.

Induction of MCD podocytes led to a significant reduction of IL-6 production and an increase in TNF-alpha in media, with concomitant higher values of IL-10, compared to unstimulated cells. Noteworthy, IL-6 was found to be the most abundant cytokine in cultured podocytes among all pro- and anti-inflammatory cytokines tested (Supp. Fig. S1C). Despite the high production of IL-6 in podocytes its value was not affected by steroid or vitamin D3 application in vitro in the MCD model. Most significant alterations were observed in reduced TNF-alpha and IL-1beta release into media in response to methylprednisolone in a dose-dependent manner, with a similar tendency demonstrated in vitamin D3-treated samples. In addition, vitamin D3 was also able to restore IFN-gamma concentration at 1000 nM towards values observed in unstimulated podocytes. A similar direction of changes was found in TGF-beta production in the presence of lower vitamin D3 concentrations. Methylprednisolone did not affect other cytokines, apart from TNF-alpha and IL-1beta, regardless of the concentration used (Fig. 4).

### Effects of vitamin D3 or methylprednisolone on MCD podocytes morphology

**Figure 5:**
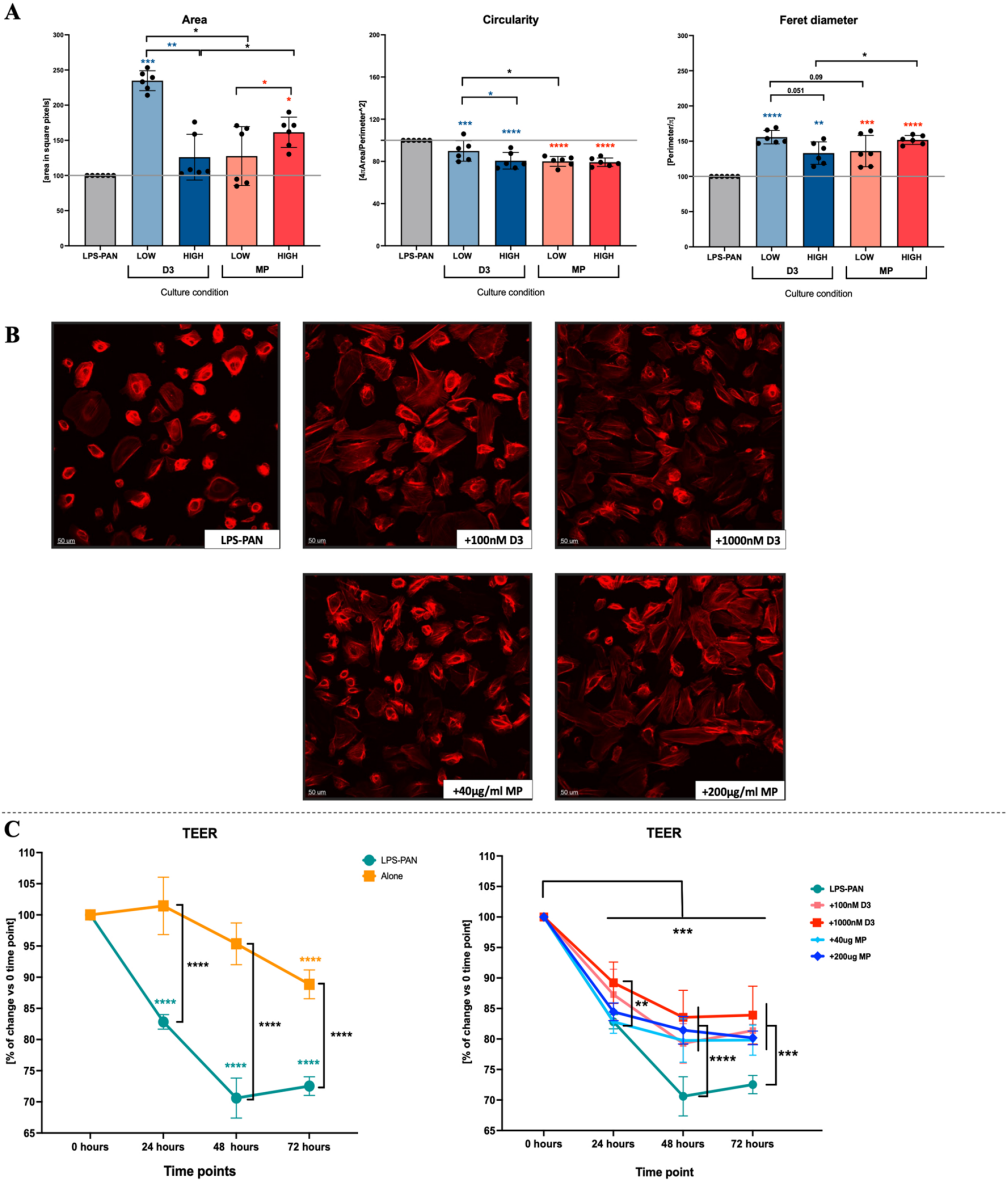
Morphological changes in podocytes subjected to different concentrations of vitamin D3 or steroid. Evaluation of podocytes area, circularity, and size (Feret diameter) in podocytes treated with vitamin D3 (D3; LOW = 100 nM, HIGH = 1000 nM) and methylprednisolone (MP; LOW = 40 μg/ml, HIGH = 200 μg/ml) (**A**). Corresponding sample data from microscopic analysis of podocytes stained with ActinRed555 for actin fibers visualization (reflecting size and shape) (**B**). Transepithelial electrical resistance (TEER) assessment in MCD in vitro model induction (left) and tested drug effects on podocytes in time (right) (**C**). Data are presented as a mean value with standard deviation (mean ± SD) (*n* = 6). The levels of significant differences were indicated with asterisks or exact p values: *p < 0.05; **p < 0.01; ***p < 0.001; ****p < 0.0001.

Induction of the MCD in vitro model through LPS-PAN stimulation of podocytes was found to reduce their area significantly, thus visibly decreasing the distance between cells. In parallel, we demonstrated higher circularity and reduced size of cells (Feret diameter) (Supp. Fig. S1E). Application of vitamin D3 led to an increase of podocytes area and size at 100 nM with the effect diminished or maintained respectively at the highest concentration. Restoration of changes in podocytes induced by MCD generation was also found in methylprednisolone-treated samples at 200 μg/ml only. Considering lower concentrations of vitamin D3 or steroid, vitamin application was associated with more pronounced effects. In accordance, vitamin D3 was found to be more effective in restoring more polymorphic shape of cells, with morphology closer to podocytes before MCD induction (Fig. 5A,B).

Results on significant changes in the morphology of podocytes treated with vitamin D3 or methylprednisolone were followed by an analysis of podocytes’ barrier function through Transepithelial electrical resistance (TEER) assessment. As expected, initial experiments revealed that morphological changes resulting from MCD induction are associated with disrupted podocyte barrier function and accompanying cellular layer electrical resistance reduction. Noteworthy, vitamin D3 predominantly effectively reduced LPS-PAN-related effects, already at 24-h incubation and 1000 nM concentration. Following 48 and 72 h of podocytes culture, all applied drugs at concentrations used managed to prevent further decline in electrical resistance. Despite not achieving initial TEER levels, vitamin D3 at 1000 nM seemed to be the most potent factor in preventing podocytes barrier dysfunction (Fig. 5C).

## Discussion

The difficulty in diagnosing and early detection of the Minimal Changes Disease is mainly caused by a lack of routine access to electron microscopy for examination that could reveal characteristic effacement of podocytes' foot processes^19^. The lack of specific non-invasive markers available during routine diagnostics delays the therapy application and, thus, leads to exacerbation and may decrease the effectiveness of the treatment. In reference to numerous diseases demonstrating inflammatory components, also including nephropathy, there is an increasing interest in the potential use of VDR and its potent ligand-active vitamin D3 (calcitriol)^11^. Most recent reports suggest a potential role of vitamin D3 in mitigating glomerulus damage; still, studies aimed at investigating the direct effects and mechanisms of that compound's action on podocytes are yet scarce^16^. Noteworthy, vitamin D3 implementation could be an attractive alternative in modulating immune responses. Thus, it could potentially support the treatment efficiency of steroid-resistant glomerulopathies, where another immunosuppressant is already used—cyclosporine A^20^.

Due to difficulties in detecting MCD at an early stage of the disease, the data on vitamin D3 or standard used steroid therapy are scarce and usually limited to later stages of kidney disorders. Here, using a well-described model of MCD, we were able to demonstrate in vitro the direct effects of vitamin D3 and methylprednisolone on a population of podocytes. We found that clinically possible to obtain concentrations of both drugs, not affecting the viability of studied cells, are 40 μg/ml for steroid and 100 nM for vitamin D3, respectively. Our data indicate that the viability of podocytes could be improved in the presence of both compounds and maintained at even higher concentrations. That is in consent with experiments with LPS-stimulated podocytes treated with vitamin D3^21^. Molecular analyses of another group focused on the level of apoptosis regulating proteins—Bcl2 and Bad in mice kidney cells, confirmed favorable effects of vitamin D3, especially in increased expression of VDR^22^.

Due to the relevance of the physiological structure and function of podocytes, numerous proteins are considered possible targets for future therapeutic approaches, especially in reference to the most challenging steroid-resistant glomerulonephropathies^4,5^. Previous studies suggested the possible consideration of nephrin as a podocyte apoptosis marker^23^. The gradually lower expression of nephrin in glomeruli has resulted in a progressive disease corresponding to FSGS (Focal Segmental Glomerulosclerosis) in the histological examination, thus can be crucial in losing podocytes functionality. Moreover, podocytes with reduced expression for nephrin showed an enhanced tendency to damage and reduced ability to regenerate^6^. Here, however, we found no significant association between nephrin and signs of podocyte injury. In diabetic nephropathy, podocin and nephrin were found to be profoundly reduced in renal biopsy samples^24^. In addition, results obtained from MCD in vitro model did not show any significant changes in these proteins' expression, with only slightly lower values for podocin coded by the *NPHS2* gene. In fact, nephrin loss is not commonly reported in all subjects diagnosed with MCD, with only around 34% of positive patients found in recent studies. Additionally, those usually were connected with a higher incidence of steroid resistance^2^. In many podocytopathies, including diabetic nephropathy, glomerulonephritis, and preeclampsia, nephrin is detected in the urine^10,24^. In fact, it was demonstrated that its level in the fluid correlates with proteinuria and podocyte injury, constituting a purposeful marker to detect early podocyte injury^20^. Furthermore, loss of nephrin reported in FSGS, can be a serviceable prediction factor of renal function in the long-term perspective of patients with MCD^2^. Additionally, reduced podocin expression was also found to correlate with the degree of proteinuria during MCD and FSGS^25^. In adult patients, reduced podocin expression is related to an acquired phenomenon rather than mutation within its gene^26^. Thus, the reduction of its expression could also indicate the degree of damage of the permeability barrier. Another protein, podocalyxin, has been confirmed to correlate positively with disease activity of numerous conditions associated with glomeruli defects, including FSGS, MN (membranous nephropathy), IgAN (IgA nephropathy)^4^. Therefore, it was hypothesized that glomerulus cells positive for podocalyxin might be more a symptom of regeneration than tissue damage. In fact, stem cell markers such as CD133 or *Oct-4* were detected on some of these cells present in the urine of patients with active FSGS, MN, and MPGN (membranoproliferative glomerulonephritis)^27,28^. In our study, higher values for cells expressing podocalyxin and mean cellular expression of that protein were reported within podocytes in vitro MCD model. Combined with the above information, these data might support increased regenerative activity within podocytes as a response to harmful factors. Phosphotyrosine, coded by the PTPRO gene, can be used as a more sensitive parameter of glomerulus damage than podocalyxin due to its presence in both podocytes and endothelial cells^29^. In an animal model of PAN-induced glomerular injury, the decreased expression of *PTPRO* was suggested to correlate with the occurrence of proteinuria^30^. In our in vitro model of MCD induced by LPS-PAN, the expression of phosphotyrosine on mRNA level also demonstrated slightly lower values. Furthermore, a mutation in the *PTPRO* gene was described by Ozaltini and considered a critical factor in changes in the podocytes structure and their foot processes effacement^30^.

Recently, vitamin D3 was shown to reverse the reduced expression of nephrin and podocin mRNA, together with CD63 or collagen-1, in a rat model of chronic kidney disease^31^. Similar results were shown in transgenic mice with human VDR over-expression, but still, these were limited to total kidney sections and not podocytes exclusively^22^. In general, all studies conducted on animal models reported significant changes within whole tissue samples, inter alia in the context of nephrin and podocin expression^18^. Therefore, we presume that reported changes do not have to be directly associated with podocytes phenotype. In our study, despite the potential improvement of podocytes survival in the presence of vitamin D3, we did not find it to increase nephrin or podocin levels as reported above. On the contrary, we found rather a reduction of these proteins in the presence of calcitriol, with higher values of nephrin exclusively limited to high doses of methylprednisolone. Interestingly, unlike reported previously, steroids and vitamin D3 were found to reduce podocin levels. Noteworthy, in our study, we tested actual protein, not only the mRNA level of these markers. In reference to other podocyte-related proteins such as podocalyxin, Verouti et al. reported restoration of that protein in calcitriol-treated cells. These changes, however, were found in glomerulus endothelial cells only (HGEC cell line), not podocytes^32^. Therefore, these data cannot be applied to podocyte function and changes in the presence of vitamin D3 or steroids we reported here. As demonstrated in our results, both calcitriol or steroid application resulted in a further decline in podocalyxin of MCD podocytes. These changes were predominantly reported on a molecular level in cells treated with vitamin D3. In reference to phosphotyrosine, the critical role of the protein was found in maintaining the integrity and selectivity of the filter membrane in nephrotic syndromes^30^. Importantly, our experiments revealed that calcitriol and methylprednisolone effectively increase phosphotyrosine on the protein level (and tendency in reference to *PTPRO* mRNA expression). These changes were more constant in vitamin D3, with gradually higher levels depending on the dosage.

Here we demonstrated that IL-6 is a dominant inflammation-related cytokine produced by podocytes among all tested. Noteworthy, the medium concentration of IL-6 was significantly reduced in LPS-PAN-induced in vitro model of MCD. That seems to be in consent with data indicating higher motility and wound healing properties of podocytes in increasing pro-inflammatory cytokine concentrations^33^. Thus, reduced IL-6 production by MCD podocytes might be closely associated with detected morphology changes and disturbance in barrier function in TEER analysis. About another inflammatory cytokine—TNF-alpha- MCD model podocytes initially demonstrated higher levels of that cytokine, which was found previously elevated in a rat model of diabetic nephropathy. The same research group reported significantly lower levels in the kidney tissues of calcitriol-treated animals^34^. Interestingly, we reduced in vitro TNF-alpha levels produced by podocytes through treatment with steroids and slightly (non-significantly) in calcitriol-stimulated cells. Similar observations were made in reference to IL-1beta levels in presence of steroids and high calcitriol concentrations. Considering the results of meta-analyses, where calcitriol was found to reduce pro-inflammatory TNF-alpha^35^, we presume that the observed previously phenomenon is not only limited to podocytes but involves other cells of glomerulus. Vitamin D3 was shown to predominantly increase the medium concentration of TGF-beta, together with such a tendency about IFN-gamma. On the contrary, other research groups demonstrated lower concentrations of IL-6 and TGF-beta in Sprague Dawley rats in the presence of calcitriol^31^. Due to a lack of information on the final concentration of vitamin D3 achieved and analyses performed on total tissue from glomeruli, a phenomenon associated with podocytes exclusively could be hidden among all other cells present.

Interestingly, in the preeclampsia-related glomeruli injury model, higher values of VEGF followed urine levels of nephrin and podocalyxin^10^. As demonstrated, VEGF plays a crucial role in maintaining the glomerular vasculature and the function of the filtration barrier^36^. Disruption of that barrier in diabetic patients was found to be associated with excessive VEGF abundance, resulting in nephrin reduction and an increase in endothelial fenestration^37^. However, in pregnancy complications—preeclampsia seems to be related to increased production of antiangiogenic factors, including sVEGFR1. It presence might contribute to sequestration and limitation of VEGF-A in circulation,^38^ possibly leading to higher values detected in the urine. On contrary to data presented by Kavuora et al.,^39^ we did not find MCD induction in vitro to decrease podocalyxin expression. Here, we found that LPS-PAN application led to higher frequencies of podocalyxin-expressing podocytes, together with the elevated mean expression of that protein within cells. In accordance, that phenomenon could also be associated with described above increased values of VEGF, contributing to disturbed barrier function. Our observations are supported by the fact that increasing levels of podocalyxin were reported in glomeruli with nephrotic stage progression^40^. Following the data of Achenbach et al.^27,28^ it is tempting to consider an increased value of podocalyxin as not a marker of podocyte morphology damage but rather as a response towards proper function restoration. It could be supported by a demonstrated decline in podocalyxin-positive cells in urine in patients with complete remission in the course of MCD^27^. However, podocalyxin interaction with cytoskeletal actin filaments was demonstrated preciously through cortactin influence^40^.

Morphological properties of podocytes in the course of MCD are essential aspects in the context of maintaining the proper function of the glomerular basement membrane (GMB). Our data indicate that dysfunction of podocytes in the LPS-PAN-induced MCD model is associated with significant changes in podocyte shape through disturbances in actin fibers. These data are consistent with animal model experiments with, inter alia, phosphotyrosine (GLEPP1) deficiency leading to a more rounded shape of affected podocytes^7^. Together with our data, the significant role of both vimentin and actin seems crucial in podocyte morphology disturbances driven by cytoskeletal alterations. A previous study demonstrated that vitamin D3 application combined with high expression of its receptor was associated with inhibition prevention of membrane thickening^22^. Noteworthy, in an isolated homogenous setting of the human MCD in vitro model, we confirmed that treatment with calcitriol could restore the morphology of podocytes. In addition, similar changes were also found in the setting, including the use of methylprednisolone. Furthermore, both vitamin D3 and steroid tested, effectively limited LPS-PAN induced damage of the filtration barrier composed of podocytes, reported in TEER assessment-based experiments. Protective properties of vitamin D3 in reference to podocytes were also suggested in the rat model of hypertension. There, the use of the compound preserved the normal histological structure of the kidneys and restored serum levels of albumin, vitamin D, and calcium^17^.

One of the most significant conclusions of our study is that we managed to demonstrate the influence of vitamin D3 and steroids exclusively within podocytes. A considerable number of previous studies on kidney glomerulus structure and function were based on analysis of whole tissue samples with no clear differentiation of what cell types of the glomerular basement membrane are responsible for the observed changes. Here, we revealed that the data attributed to all cells constituting the filtration barrier could not be directly related to the podocytes. Those include inter alia changes within nephrin or podocin in response to calcitriol treatment, which in our study were not found to be such significant or had completely different directions in reference to podocytes. Our assumption on the different responses of podocytes in models of nephropathies treated with vitamin D3 or steroids is supported by data from other researchers focusing exclusively on glomerular endothelial cells^32^. Based on their results and our data, we presume that previously reported changes within kidney tissue in nephropathic models, also treated with indicated compounds, could be predominantly linked to the endothelial cells of the filtration barrier and not podocytes. Here, we limited our considerations exclusively to podocytes and their role in MCD. Importantly, we revealed possible options for studied cells phenotype and function modulation through immunomodulators—active vitamin D3 and methylprednisolone. Noteworthy, despite significant results, presented data should be interpreted with caution considering the use of immortalized cell line—constituting one of the study limitations. Nonetheless, our data constitute a significant basis for future studies on tested immunosuppressants use in podocyte-related phenomenon accompanying minimal change disease.

## Materials and methods

### Podocyte cell line maintenance and MCD in vitro model induction

Conditionally immortalized human podocyte cell line CIHP-1 (constructs used for immortalization: tsSV40 and hTERT) was obtained under Material Transfer Agreement (MTA) from the University of Bristol^41^. Handling of the cell line was performed in accordance with previously published instructions, including the use of RPMI-1640 medium supplemented with fetal bovine serum (PAN Biotech), Insulin-Transferrin-Selenium reagent (Gibco), and gentamicin (Gibco); and culture at 33 °C in 5% CO_2_^42^. When the appropriate number was obtained, cells were transferred into experimental culture plates and incubated further at 33 °C, 5% CO_2_, until reaching 75–85% confluence. Media has been replaced, and from that moment, cells were cultured at 37 °C, 5% CO_2_, to allow their differentiation towards mature podocytes. Generated podocytes were subjected to induction of MCD in vitro model in accordance with previously published protocols,^43^ modified and verified to obtain essential efficiency. Here, we used 25 μg/ml lipopolysaccharide (LPS; ultra-pure lipopolysaccharide from E. coli 0111: B4 strain, InvivoGen) and 60 μg/ml of puromycin aminonucleoside (PAN; Cayman Chemicals), for 24 h, to generate the cells morphology characteristic for MCD. Separate analyses were performed to evaluate LPS and PAN effects on podocytes in vitro (Supp. Fig. S1). The implication of PAN is a common method for MCD induction in animal models. Spontaneous incidence of that condition was only reported in Sprague Dawley rats^44^. In vitro model using CIHP-1 cells is an animal-free alternative to the MCD model, allowing for recreating MCD podocyte characteristics in a laboratory setting. Despite reported influence of the induction process on the podocyte markers, the validity of that immortalized podocytes model is preserved and is commonly implemented in various studies^45^. No animals or patients’ materials were used in the described experiments.

### MCD model podocytes incubation with vitamin D3 or methylprednisolone

Podocytes obtained after MCD model induction with LPS and PAN were used to study vitamin D3 and steroid use on these cells. Active form of vitamin D—calcitriol, was used at a concentration of 100 nM and 1000 nM (calcitriol, 1,25-dihydroxycholecalciferol; Cayman Chemicals), whereas steroid—methylprednisolone, concentration was implemented at 40 μg/ml and 200 μg/ml (methylprednisolone sodium succinate; Solu-Medrol, Pfizer). Following 48-h incubation at 37 °C, 5% CO_2_, materials were collected for further analyses, including culture supernatants (stored at − 80 °C) and viable cells for flow cytometric analysis. Additional portion of podocytes was subjected to treatment with RLT buffer (Qiagen) supplemented with β-mercaptoethanol (Sigma-Aldrich) for genetic material isolation (stored at − 80 °C). Each experiment was performed in 10 separate repetitions.

### Flow cytometric analysis of viability and podocyte-related proteins

Podocytes collected after cell culture and washed in phosphate-buffered saline (PBS; Corning) were distributed into dedicated tubes and stained with monoclonal antibodies: anti-Phosphotyrosine AlexaFluor488 (clone PY20; Biolegend), anti-Podocalyxin AlexaFluor647 (clone TRA-1-81; Biolegend), anti-Nephrin (polyclonal; Invitrogen), anti-Podocin/NPHS2 (polyclonal; MyBiosource). For staining of the intracellular protein—Phosphotyrosine, prior incubation with FACS Permeabilization 2 buffer was required (BD Bioscience). Incubation with antibodies for 25 min (room temperature, dark) was followed by washing steps with PBS. In reference to anti-Nephrin and anti-Podocin antibodies, staining with secondary fluorochrome-conjugated antibodies was applied using: goat anti-rabbit AlexaFluor546 (polyclonal; Invitrogen), goat anti-rabbit AlexaFluor488 (polyclonal; Invitrogen). After 25 min (room temperature, in the dark), cells were washed twice in PBS and suspended in fixation buffer (CellFIX; BD Bioscience). FACS Calibur (BD Bioscience; San Jose, CA, USA) device was used for data acquisition. Data processing was performed with FlowJo software (TreeStar Inc.; Ashland, OR, USA). The gating strategy for the proteins assessment involved initial podocyte morphology determination—size (forward scatter, FSC) and granularity/complexity (side scatter, SSC), and baseline selection based on FMO (fluorescence-minus one) and negative controls.

Viability assessment protocol involved the suspension of the cells in Annexin V Binding Buffer (Biolegend) and incubation of cells in the presence of APC-conjugated Annexin V and 7-aminoactinomycin D (7AAD) (Biolegend) for 15 min, in dark, room temperature. Data were acquired within 1 h on a flow cytometer and analyzed with FlowJo software. Gating strategy based on changes in 7AAD and Annexin V expression allowed for a determination of: viable (7AAD−Annexin-V−), early apoptotic (7AAD−Annexin-V +), late apoptotic (7AAD + Annexin-V +) and necrotic (7AAD + Annexin-V−) podocytes. Gating strategy for podocyte-related proteins and viability has been included within supplementary materials (Supp. Fig. S2).

### Expression analysis of selected podocyte-related proteins on mRNA level

Podocytes genetic material was subjected to RNA extraction with the use of spin columns of the RNeasy Micro kit (Qiagen). The obtained material's concentration and purity were confirmed on the NanoDrop 2000 spectrophotometer (Thermo Fisher Scientific; Waltham, MA, USA). Absorbance was measured at 260 nm and 280 nm, setting the range for pure material at 1.8–2.2 for A260/A280 ratio. cDNA was generated from the RNA using High-Capacity cDNA Reverse Transcription kit (Thermo Fisher) by following steps: (10 min, 25 °C), (120 min, 37 °C), (5 min, 85 °C). For each repeat of selected gene expression analysis 10 ng of cDNA was used in mRNA assessment. Subsequent RT-PCR analyses were performed using mentioned genetic material, TaqMan Universal Master Mix (containing AmpliTaq Gold DNA Polymerase, Uracil-DNA Glycosylase, dNTPs with dUTP, Passive Reference 1 and optimized buffer components) (Qiagen), and selected Gene Expression Assays (FAM) (TaqMan; Thermo Fisher). For detection of the podocyte-related proteins, mRNA TaqMan probes were used: *NPHS1* (nephrin) (assay ID: Hs00190446_m1; spanned exons 22 to 23), *NPHS2* (podocin) (assay ID: Hs00387817_m1; spanned exons 1 to 2), *PTPRO* (Phosphotyrosine) (assay ID: Hs00958177_m1; spanned exons 13 to 14), *PODXL* (Podocalyxin) (assay ID: Hs01574644_m1; spanned exons 7–8). In addition, *GAPDH* (glyceraldehyde-3-phosphate dehydrogenase) (assay ID: Hs02786624_g1; within exon 7) was used as a housekeeping gene. Conditions for RT-PCR analysis were: 1 cycle of UNG incubation (50 °C, 2 min), 1 cycle of enzyme activation (95 °C, 10 min), 40 cycles of denaturation (95 °C, 15 s) and annealing with elongation (60 °C, 1 min). The whole process was performed using StepOnePlus Real-Time PCR System device (Applied Biosystems; Foster City, CA, USA), and obtained data were analyzed with the attached dedicated software (StepOnePlus software v2.3).

### Immunoenzymatic assessment of cytokines produced by MCD model podocytes

Supernatants from cell cultures of podocytes were assessed in the context of selected pro- and anti-inflammatory cytokines release. Commercially available immunoenzymatic ELISA kits implementation allowed for the detection of IL-1β, IL-6, IL-10, TGF-β, TNF-alpha, IFN-γ (DuoSet; R&D Systems). All the procedures were performed in accordance with the kit protocols. Data from prepared plates were acquired using LEDETECT96 microplate reader (Labexim Products, Lengau, Austria), reading absorbance at 450 nm, with background correction set at 620 nm. The concentration of the cytokines was established based on a standard curve using a four-parameter logistic (4-PL) curve fit.

### Confocal microscope analysis of morphological features of tested podocytes

The podocyte’s morphology was visualized using the FV1200 confocal microscope (Olympus; Hamburg, Germany). Determination of changes in cells shape and area was implemented through staining of the actin fibers with ActinRed555 reagent (Thermo Fisher)—F-actin probes (phalloidin) conjugated to fluorescent tetramethylrhodamine (TRITC). Following photos acquisition, exact measurements of podocytes area, circularity (circularity = 4π(area/perimeter^2^); 1.0—a perfect circle, values towards 0.0—elongated polygon), and size (Feret diameter = longest distance between any two points along the selection boundary) were performed using ImageJ software^46^.

### Investigation of transepithelial electrical resistance (TEER) of podocytes

In the context of maintaining an effective filtration membrane, podocyte layer integrity was analyzed with noninvasive measurement of transepithelial/epithelial electrical resistance (TEER). Podocytes have been seeded on cell culture inserts with a pore size of 0.4 μm (Greiner Bio-One) and incubated until complete coverage of the surface. Cells layer was tested according to the previous points' conditions at three-time points (24, 48 and 72 h). The electrical resistance of the cell layer (presented in ohms, Ω) was performed with the STX2-Plus electrode combining two electrodes for current sourcing and two for voltage measurement. Resistance was automatically calculated in accordance with the equation: R = U/I (R—resistance, V—voltage, I—current) and subtraction of the blank resistance measurement (porous membrane in medium only). The presented evaluation was implemented using an epithelial volt-ohm meter 3 device (EVOM3; World Precision Instruments, Sarasota, FL, USA).

### Biostatistical analysis of the data

Statistical processing of the data collected was performed with GraphPad Prism software (GraphPad Prism Software Inc., San Diego, CA, USA). Effects of different drug concentrations tested compared to podocytes alone and differences in response between them were analyzed using two-way ANOVA with Fisher’s LSD test for multiple comparisons. Data are presented as a mean value with standard deviation (mean ± SD). The statistical significance level was set at p < 0.05, and significant changes were indicated with asterisks or exact p values for tendencies: *p < 0.05, **p < 0.01, ***p < 0.001, ****p < 0.0001. To focus data visualization on responses to in vitro stimulations, data were presented as percentage changes of individual values in reference to podocytes alone (LPS-PAN stimulated only), referred to as 100%.

### Informed consent

No animals or patients’ materials were used in the described experiments.

## Conclusions

One of the most significant conclusions of our study is that we managed to demonstrate the influence of active vitamin D3 and steroids exclusively on podocytes. A considerable number of previous studies on kidney glomerulus structure and function were based on analysis of whole tissue samples with no clear differentiation of what cell types of the glomerular basement membrane are responsible for the observed changes. Here, we revealed that the data reported so far attributed to all cells constituting the filtration barrier could not be directly related to the podocytes. Those include inter alia changes within nephrin or podocin in response to calcitriol treatment, which in our study were not found to be such significant or showed completely different directions in reference to podocytes. Our assumption on the different responses of podocytes in models of nephropathies treated with vitamin D3 or steroids is supported by data from other researchers focusing exclusively on glomerular endothelial cells^32^. Based on their results and our data, we presume that previously reported changes within kidney tissue in nephropathic models, also treated with indicated compounds, could be predominantly linked to the endothelial cells of the filtration barrier and not podocytes. Here, we limited our considerations exclusively to podocytes and their role in MCD. Importantly, we revealed possible options for studied cells phenotype and function modulation through immunomodulators—calcitriol and methylprednisolone. Noteworthy, despite significant results, presented data should be interpreted with caution considering the use of immortalized cell line—constituting one of the study limitations. Nonetheless, our data constitute a significant basis for future studies on tested immunosuppressants use in podocyte-related phenomenon accompanying minimal change disease.

## Future directions

Undoubtedly, our research results presented a new approach in using immunosuppressive compounds in the context of minimal change disease. Further in-depth research is of great interest and will certainly contribute not only to the development of effective methods of supporting conventional therapies. In accordance, more detailed insight into the involvement of podocytes is required in the context of damage of the filtration barrier, as well as pathways responsible for its restoration with implementation of immunomodulatory compounds. Considering our research, those should predominantly include active vitamin D3—calcitriol, and its combination with steroid used commonly in clinical setting.

### Supplementary Information

Supplementary Figures.

## Data Availability

The datasets used and/or analysed during the current study available from the corresponding author on reasonable request.
