# Supplementary material for: The influence of calcitriol and methylprednisolone on podocytes function in minimal change disease in vitro model
Source: Sci Rep. 2023 Aug 5;13:12731. doi: 10.1038/s41598-023-39893-x (PMC10404287; doi:10.1038/s41598-023-39893-x)
Supplement: Supplementary file 1 — Supplementary Figures. [file 41598_2023_39893_MOESM1_ESM.pdf]

“The influence of calcitriol and methylprednisolone on podocytes function in Minimal Change Disease in vitro model.”

Supplementary Figure 1

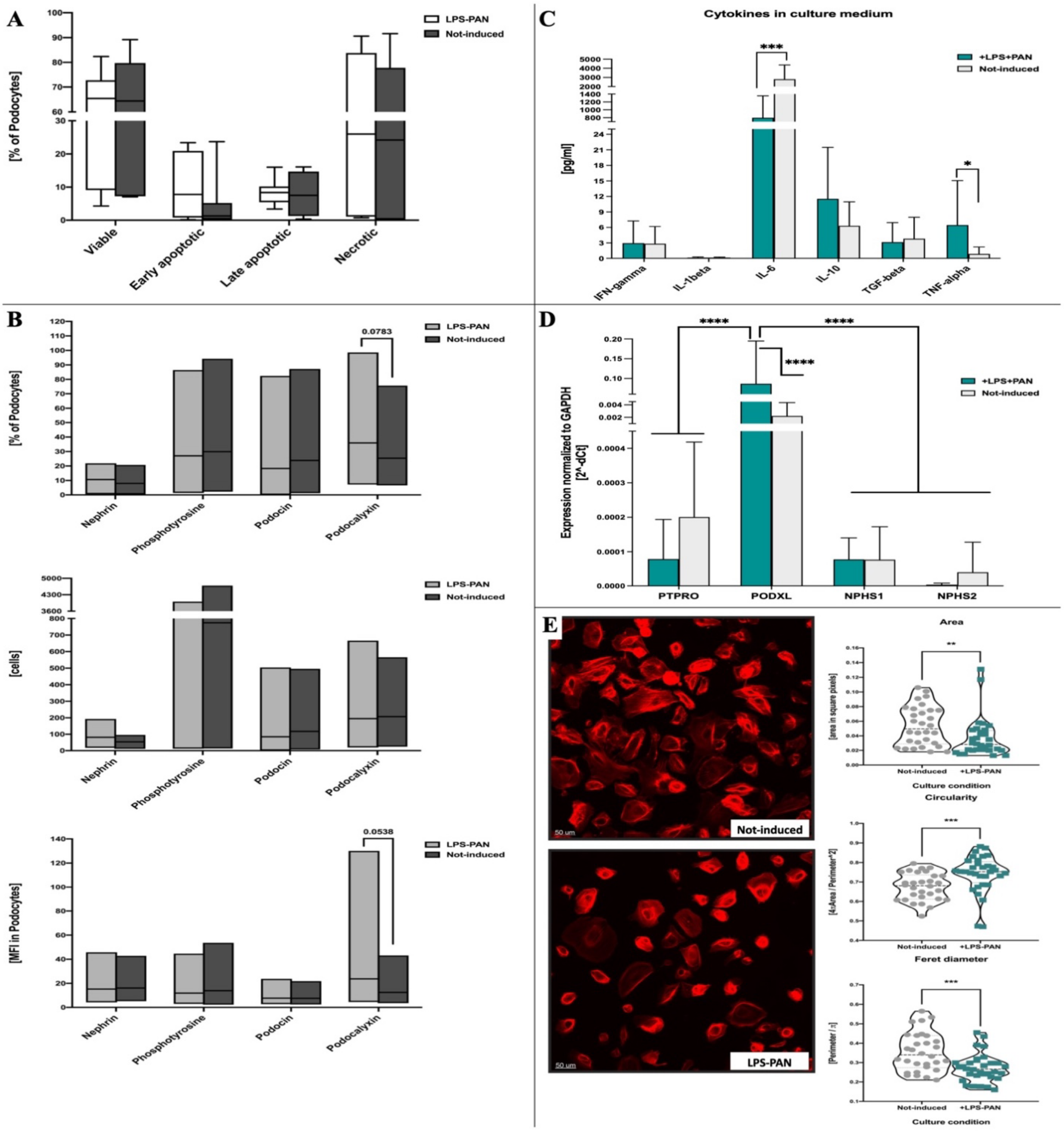

Supplementary Figure 1. Characterization of podocytes in response to LPS-PAN induction in MCD in vitro model. AnnexinV- and 7-AAD-based viability assessment within podocytes in response to LPS-PAN induction (A). Expression of selected podocyte-related proteins after incubation with LPS-PAN, including frequency of cells, absolute numbers, and mean fluorescence intensity (MFI) (B). Evaluation of selected cytokines released into the media by podocytes subjected to LPS-PAN stimulation (C). Analysis of genes related to tested podocyte proteins in LPS-PAN treated podocytes: nephrin (*NPHS1*), phosphotyrosine (*PTPRO*), podocin (*NPHS2*), and podocalyxin (*PODXL*) (D). Assessment of changes in podocyte area, circularity and size (Feret diameter) in response to MCD induction with LPS-PAN (E). The levels of significant differences were indicated with asterisks or exact p values: * - $p < 0.05$; ** - $p < 0.01$; *** - $p < 0.001$; **** $p < 0.0001$.

Supplementary Figure 2

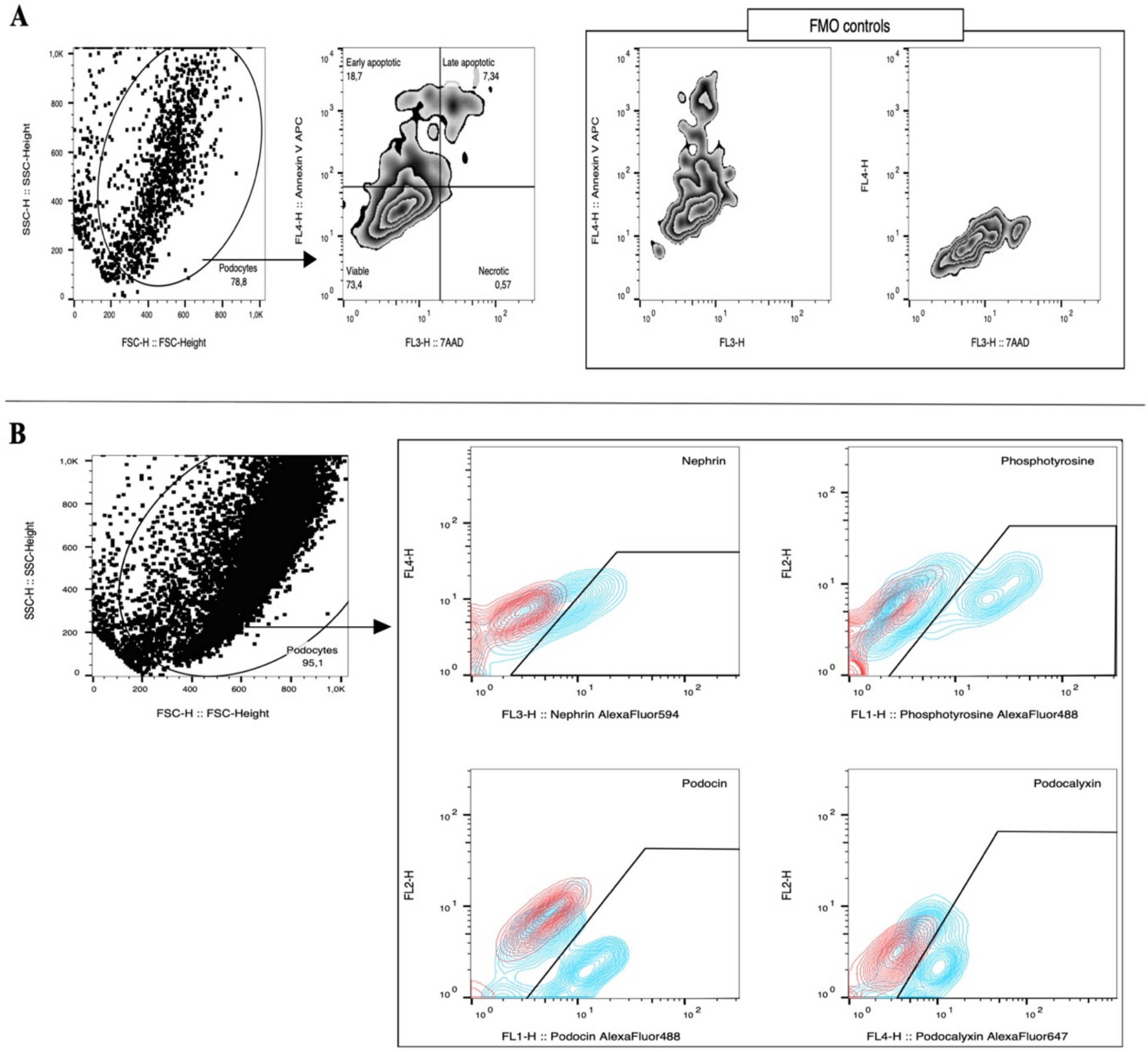

Supplementary Figure 2. Gating strategy of podocytes implemented in *in vitro* MCD model. Assessment of viability with 7AAD and AnnexinV APC staining (A). Evaluation of nephrin, phosphotyrosine, podocin (NPHS-2), and podocalyxin (TRA-1-81) within podocytes (B).

Supplementary Figure Legends

Supplementary Figure 1. Characterization of podocytes in response to LPS-PAN induction in MCD in vitro model. AnnexinV- and 7-AAD-based viability assessment within podocytes in response to LPS-PAN induction (A). Expression of selected podocyte-related proteins after incubation with LPS-PAN, including frequency of cells, absolute numbers, and mean fluorescence intensity (MFI) (B). Evaluation of selected cytokines released into the media by podocytes subjected to LPS-PAN stimulation (C). Analysis of genes related to tested podocyte proteins in LPS-PAN treated podocytes: nephrin (*NPHS1*), phosphotyrosine (*PTPRO*), podocin (*NPHS2*), and podocalyxin (*PODXL*) (D). Assessment of changes in podocyte area, circularity and size (Feret diameter) in response to MCD induction with LPS-PAN (E). The levels of significant differences were indicated with asterisks or exact p values: * - $p < 0.05$; ** - $p < 0.01$; *** - $p < 0.001$; **** $p < 0.0001$.

Supplementary Figure 2. Gating strategy of podocytes implemented in *in vitro* MCD model. Assessment of viability with 7AAD and AnnexinV APC staining (A). Evaluation of nephrin, phosphotyrosine, podocin (NPHS-2), and podocalyxin (TRA-1-81) within podocytes (B).
